# Autoimmune glial fibrillary acidic protein astrocytopathy coexistent with reversible splenial lesion syndrome: A case report and literature review

**DOI:** 10.3389/fneur.2023.1192118

**Published:** 2023-05-25

**Authors:** Jing Lin, Liangbin Dong, Li Yu, Jingwei Huang

**Affiliations:** Department of Neurology, The First Affiliated Hospital of Nanchang University, Nanchang, China

**Keywords:** autoimmune glial fibrillary acidic protein astrocytopathy, reversible splenial lesion syndrome (RESLES), magnetic resonance imaging, linear perivascular radial enhancement, leptomeningeal enhancement

## Abstract

Autoimmune glial fibrillary acidic protein (GFAP) astrocytopathy is a rare autoimmune disorder. Reversible splenial lesion syndrome (RESLES) is a transient clinical-imaging syndrome characterized by specific magnetic resonance imaging (MRI) pattern. A 58-year-old man was admitted with a fever, headache, and confusion for 1 week. Brain MRI showed abnormal leptomeningeal enhancement in the brainstem and high signal intensity on diffusion-weighted MRI of the corpus callosum. Anti-GFAP antibody was positive in the serum and cerebrospinal fluid analysis. This patient significantly improved and had no relapse after glucocorticoid and immune suppressant therapy. A repeated brain MRI revealed the lesion in the corpus callosum and abnormal leptomeningeal enhancement in the brainstem disappeared. Linear perivascular radial enhancement is the characteristic pattern of autoimmune GFAP astrocytopathy which is rarely coexistent with RESLES.

## Introduction

Autoimmune glial fibrillary acidic protein (GFAP) astrocytopathy, first described in 2016, is an immune-mediated central nervous system disorder characterized by GFAP antibody (1). This disease mainly involves the meninges, spinal cord, and cerebra, and its predominant clinical syndrome include encephalopathy, myelitis, fever, headache, and epilepsy (2). The characteristic imaging feature of autoimmune GFAP astrocytopathy is linear perivascular radial gadolinium enhancement, but its diagnosis mainly depends on GFAP-IgG in cerebrospinal fluid (2).

Reversible splenial lesion syndrome (RESLES) is a transient clinical-imaging syndrome characterized by the presence of a focal lesion often involving the corpus callosum (3). RESLES was first described to be triggered by antiepileptic drugs, and then it has been reported to be secondary to a variety of factors, including infection, metabolic disorders, and autoimmune diseases (4, 5).

Herein, we report an autoimmune GFAP astrocytopathy concurrent with RESLES in an adult patient. Additionally, we discuss the imaging features of autoimmune GFAP astrocytopathy, with respect to the results of previous studies.

## Case presentation

**Figure 1:**
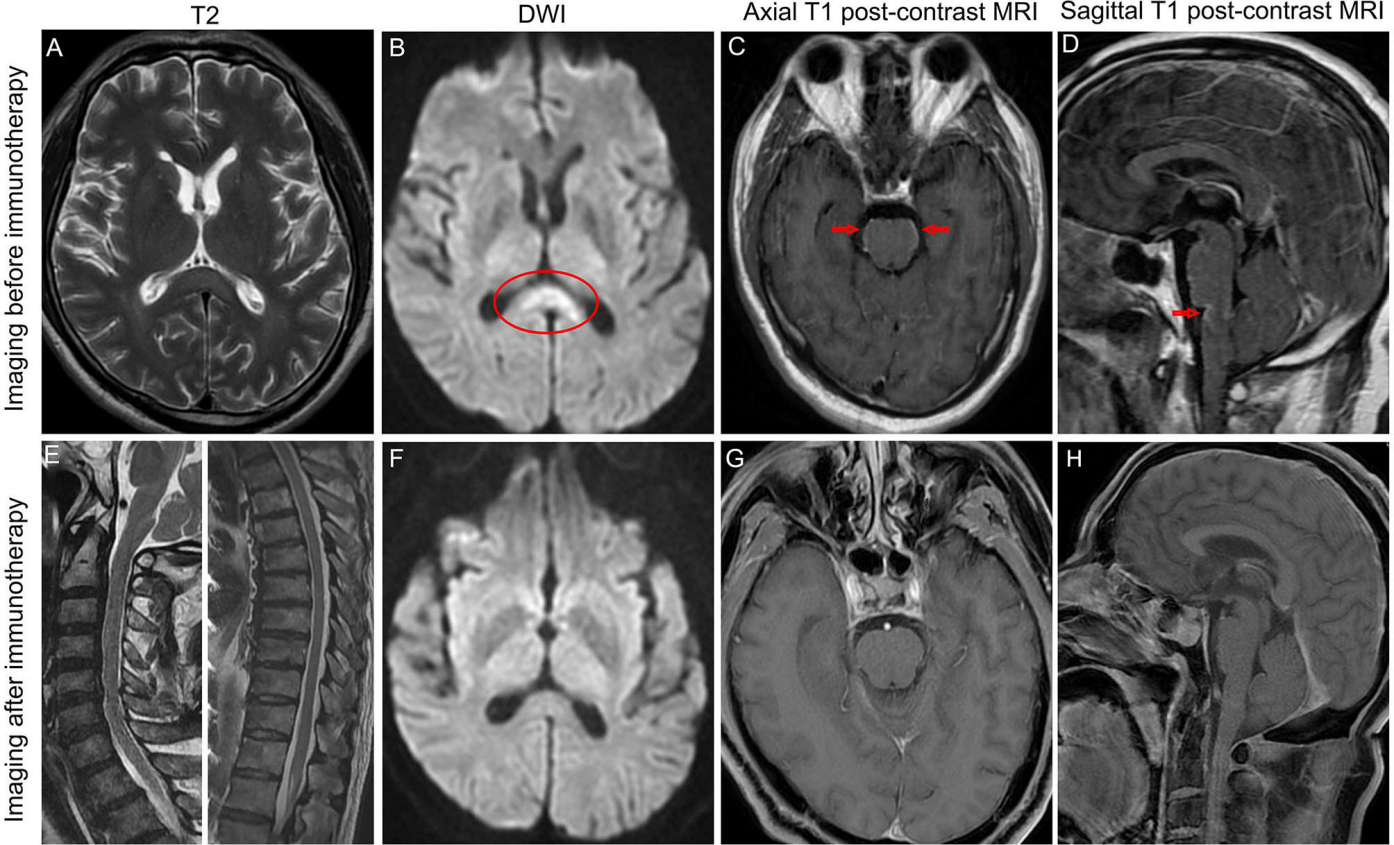
Brain MRI revealed hyperintense of the corpus callosum on T2 sequence MRI **(A)** and diffusion-weighted MRI **(B)** before immunotherapy. MRI postcontrast axial T1-weighted scans **(C)** and sagittal T1-weighted scans **(D)** showed leptomeningeal enhancement of the pons and medulla before immunotherapy. Sagittal T2 sequence MRI showed no hyperintensities throughout the cervical spinal cord and thoracic spinal cord **(E)**. The high signal intensity of the corpus callosum on diffusion-weighted MRI disappeared after immunotherapy **(F)**. MRI postcontrast axial T1-weighted scans and sagittal T1-weighted scans showed that leptomeningeal enhancement of the pons and medulla completely disappeared after immunotherapy **(G, H)**. Arrows indicate leptomeningeal enhancement. Oval shape indicates the lesion in the corpus callosum.

A 58-year-old man presented with a fever (39.8°C), headache, and confusion for 1 week. He has no previous history of other illnesses or medication use. A neurological examination demonstrated neck stiffness, positive Kernig's sign, and motor power grade 4 in both lower extremities. Blood routine examination, C-reactive protein, and procalcitonin were in the normal range. The cerebrospinal fluid (CSF) sample displayed normal open pressure (110 mmH2O), slightly elevated nucleated cells of 80/ul (normal range: 0–10/ul), significantly elevated protein of 158.71 mg/L (normal range: 15–45 mg/dL), and decreased glucose level of 2.15 mmol/L (blood glucose: 7.29 mmol/L). Brain magnetic resonance imaging (MRI) revealed abnormal leptomeningeal enhancement in the brainstem (Figures 1C, D). In addition, brain MRI showed the high signal intensity of the corpus callosum on T2 sequence MRI (Figure 1A) and diffusion-weighted MRI (Figure 1B). Possible tuberculous meningoencephalitis was diagnosed and anti-tuberculosis therapy was started with oral isoniazid, rifampicin, pyrazinamide, and ethambutol.

A few days after admission, acute delirium and urinary retention developed. A repeated lumber puncture showed high open pressure (250 mmH2O), normal nucleated cells, significantly elevated protein (119.13 mg/L), and decreased glucose level (2.65 mmol/L).

**Figure 2:**
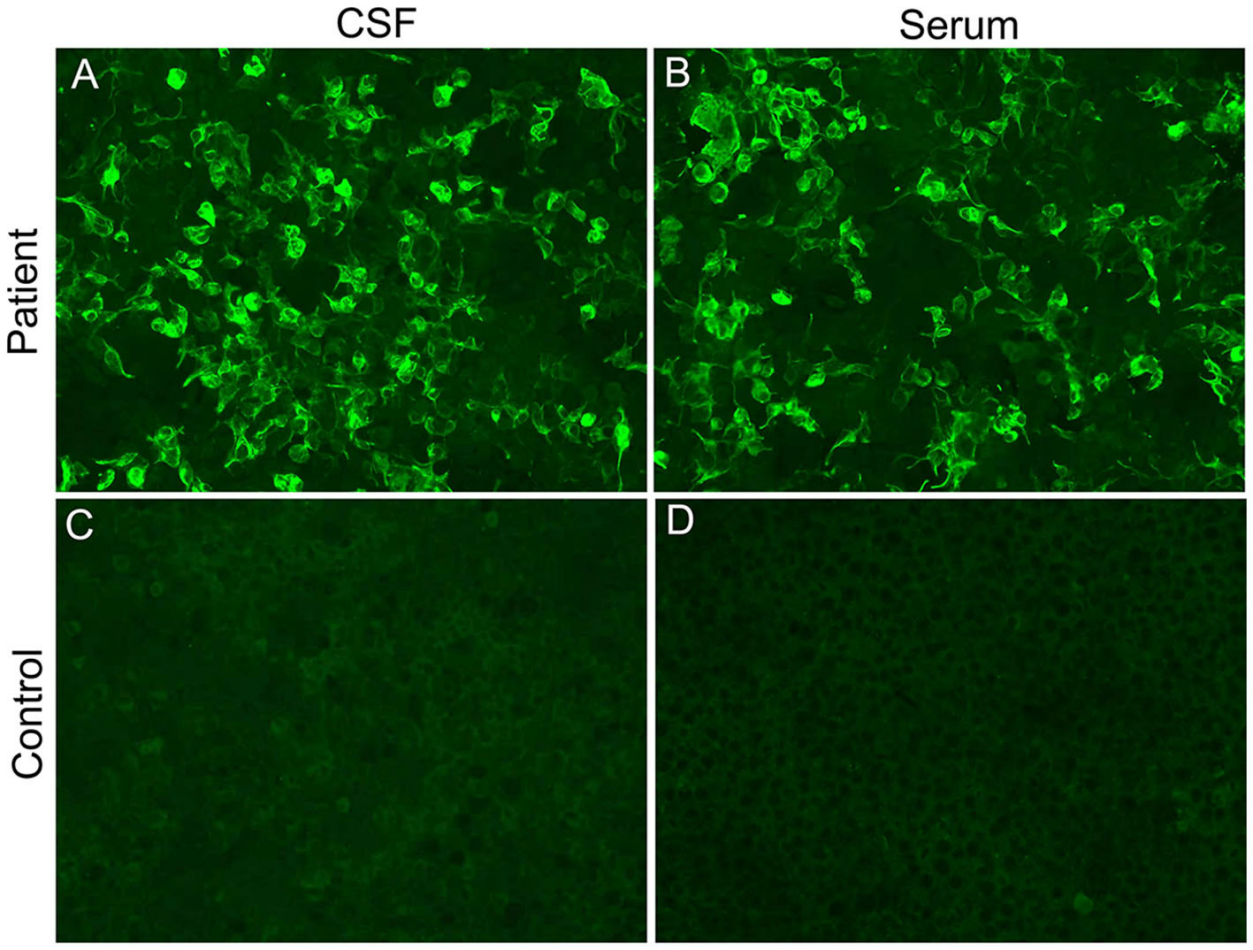
Cell-based indirect immunofluorescence assays demonstrating positive GFAPα-immunoglobulin G in CSF **(A)** and serum **(B)** from our patient. **(C, D)** represent negative controls.

On the 13th day after admission, the patient's condition deteriorated again, with light coma (remaining light reflex and responding to pain) and epilepsy. The neurological examination revealed motor power grade 0 in both lower extremities, tendon areflexia, and limited bilateral abduction. Second-generation sequencing (BGI platform, China) for CSF in this patient revealed no abnormalities. We sent an autoimmune encephalitis panel and demyelinating antibody panel including serum and CSF samples, and the anti-GFAP antibody was positive (1:10) in the serum and CSF analysis (Figure 2). Subsequently, anti-tuberculosis therapy was discontinued and the patient was treated with intravenous 500 mg methylprednisolone per day for 5 days, 240 mg per day for 5 days, 120 mg per day for 5 days, and followed by a slow tapering dose of prednisone over half year. Additionally, the patient received oral immune suppressants (mycophenolate mofetil 500 mg twice daily) for 6 months.

On the fifth day after intravenous methylprednisolone, the patient's consciousness and motor power improved significantly. After completing glucocorticoid pulse therapy, all symptoms of the patient disappeared except urinary retention, and a repeat brain MRI revealed the lesion in the corpus callosum and abnormal leptomeningeal enhancement in the brainstem disappeared (Figures 1F–H). In addition, the spine MRI showed no T2 hyperintensities throughout the cervical spinal cord and thoracic spinal cord (Figure 1E). For urinary retention, we give oral vitamins B1 and B12, and acupuncture and moxibustion rehabilitation training. At follow-up 1 year after symptom onset, the urinary retention of our patient partly improved, and the patient remained clinically stable and had no relapse.

## Discussion

Reversible splenial lesion syndrome (RESLES) is a rare clinical-imaging syndrome involving the corpus callosum caused by various causes (3). It is characterized by oval, non-enhanced T2 lesions in the splenium of the corpus callosum on MRI, which can completely disappear after a variable period of time (3). This syndrome was related to a variety of factors including antiepileptic drugs, infections, hypoglycemia, hyperglycemia, and electrolyte imbalance (4, 5). Recently, a potential association between autoimmune processes and RESLES has been documented in cases such as N-methyl-D-aspartate receptor encephalitis (6), autoantibodies against voltage-gated potassium channels (7), autoimmune thyroid disease (8), and so on. Herein we report a rare case of autoimmune GFAP astrocytopathy coexistent with RESLES.

Currently, there are no uniform diagnostic criteria or consensus for autoimmune GFAP astrocytopathy. The diagnosis of autoimmune GFAP astrocytopathy may be based on the presence of GFAP antibodies in the CSF and clinical scpectrum (9). The classical phenotype of autoimmune GFAP astrocytopathy is meningoencephalitis or myelomeningoencephalitis but the clinical manifestations are broader, including fever, headache, delirium, seizures, tremor, and so on (2). Our patient presented with fever, headache, confusion, seizures, and myelomeningoencephalitis. Additionally, the initial CSF results suggested tuberculous meningoencephalitis, and then anti-tuberculosis therapy was started. However, an anti-GFAP antibody was detected positive in the serum and CSF analysis in our patient. Furthermore, we excluded autoimmune encephalitis and central nervous system infection through negative autoimmune encephalitis antibodies and negative second-generation sequencing in this patient. Taken together, this patient should be diagnosed with autoimmune GFAP astrocytopathy, and anti-tuberculosis therapy was discontinued.

Our search of the PubMed database focusing on autoimmune GFAP astrocytopathy yielded 10 studies that reported more than 10 cases. The imaging profiles of these reports are summarized in Table 1. The most frequent brain MRI finding of autoimmune GFAP astrocytopathy was hyperintense lesions in the cerebra on T2 sequence such as basal ganglia, thalamus, cerebral white matter, brainstem, and so on. However, it has been reported linear perivascular radial enhancement is a hallmark of autoimmune GFAP astrocytopathy and was observed in 42.3% of patients. Moreover, 29.8% of patients had leptomeningeal enhancement. Longitudinally extensive transverse myelitis (more than three vertebra segments) was the most common finding in autoimmune GFAP astrocytopathy, followed by enhancement of medullary cone surface and intramedullary enhancement. More importantly, autoimmune GFAP astrocytopathy coexistent with RESLES was rarely reported, only one study has summarized this image feature and 4 out of 39 autoimmune GFAP astrocytopathy patients had RESLES (17). Furthermore, four recent cases who had autoimmune GFAP astrocytopathy coincident with RESLES were reported (19–22). However, five sporadic cases including our patient with autoimmune GFAP astrocytopathy and RESLES showed no obvious imaging association between linear perivascular radial enhancement and RESLES. Additionally, RESLES had been reported in cases of anti-Yo encephalitis (23), anti-NMDAR encephalitis (24), and anti-VGKC encephalitis (7), respectively. Taken together with the findings from previous case series, our present findings suggested RESLES may be linked to autoimmune processes and is rarely coexistent with autoimmune GFAP astrocytopathy.

**Table 1:** The imaging summarization of autoimmune GFAP astrocytopathy patients.

	**Fang et al. (1)**	**Flanagan et al. (10)**	**Dubey et al. (11)**	**Iorio et al. (12)**	**Long et al. (13)**	**Yang et al. (14)**	**Kimura et al. (15)**	**Fang et al. (16)**	**Gravier et al. (17)**	**Liao et al. (18)**	**Total**
**Brain MRI**											
Hyperintense lesions on T2WI/FLAIR	9/12 (75%)	18/32 (56%)	NA	10/22 (45%)	8/19 (42.1%)	9/10 (90%)	9/14 (64%)	30/35 (85.7%)	20/39 (51.3%)	13/15 (86.6%)	126/198 (63.6%)
Linear perivascular radial enhancement	6/12 (50%)	17/32 (53%)	36/71 (51%)	NA	8/19 (42.1%)	4/10 (40%)	4/9 (44.4%)	3/35 (8.5%)	12/38 (31.6%)	12/15 (80%)	102/241 (42.3%)
Leptomeningeal enhancement	4/12 (33%)	7/32 (22%)	NA	NA	NA	NA	3/9 (33.3%)	6/35 (17.1%)	10/38 (26.3%)	12/15 (80%)	42/141 (29.8%)
RESLES	NA	NA	NA	NA	NA	NA	NA	NA	4/39 (10.3%)	NA	4/39 (10.3%)
**Spinal MRI**											
Longitudinally extensive T2 hyperintensity	5/7 (71%)	6/8 (75%)	NA	3/9 (33%)	11/16 (68.8%)	6/9 (66.7%)	NA	5/35 (14.3%)	9/11 (81.2%)	5/15 (33.3%)	50/110 (45.5%)
Intramedullary enhancement	NA	NA	NA	NA	NA	NA	1/7 (14%)	3/35 (8.5%)	4/11 (36.4%)	7/15 (46.7%)	15/68 (22.1%)
Enhancement of medullary cone surface	NA	NA	NA	NA	NA	NA	5/7 (71%)	3/35 (8.5%)	4/11 (36.4%)	9/15 (60%)	21/68 (32.3%)

GFAP, glial fibrillary acidic protein; MRI, magnetic resonance imaging; NA, not available; FLAIR, fluid-attenuated inversion-recovery sequency; RESLES, reversible splenial lesion syndrome.

## Conclusion

In summary, the most frequent brain MRI finding of autoimmune GFAP astrocytopathy was hyperintense lesions in the cerebra on the T2 sequence. However, linear perivascular radial enhancement is a hallmark of autoimmune GFAP astrocytopathy. Autoimmune GFAP astrocytopathy coexistent with RESLES is rare.

## Data availability statement

The raw data supporting the conclusions of this article will be made available by the authors, without undue reservation.

## Ethics statement

The studies involving human participants were reviewed and approved by the Ethics Committee of The First Affiliated Hospital of Nanchang University. The patients/participants provided their written informed consent to participate in this study. Written informed consent was obtained from the individual(s) for the publication of any potentially identifiable images or data included in this article.

## Author contributions

JL and JH contributed to the conception and design of the study. JL wrote the initial draft of the manuscript. LD and LY contributed to the clinical analysis. JH contributed to the manuscript's revision. All authors read and approved the submitted version.
